# Supplementary material for: Locked in Structure: Sestrin and GATOR—A Billion-Year Marriage
Source: Cells. 2024 Sep 21;13(18):1587. doi: 10.3390/cells13181587 (PMC11429811; doi:10.3390/cells13181587)
Supplement: Supplementary file 1 [file cells-13-01587-s001.zip › Supplement File S1 - MEME discovery.docx]

| Metazoan 6 residues wide |  |
| --- | --- |
| MEME discovered motif | Conservation (N=1006) |
| 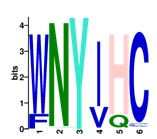 | \| 394 \| 395 \| 396 \| 397 \| 398 \| 399 \| \| --- \| --- \| --- \| --- \| --- \| --- \| \| W \| N \| Y \| I \| H \| C \| \| W \| N \| Y \| I \| H \| C \| \| 84 \| 97 \| 100 \| 74.5 \| 84.2 \| 92.7 \| \| **845** \| **976** \| **1006** \| **749** \| **847** \| **933** \| |
| 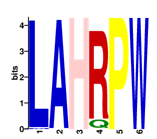 | \| 166 \| 167 \| 168 \| 169 \| 170 \| 171 \| \| --- \| --- \| --- \| --- \| --- \| --- \| \| L \| A \| H \| R \| P \| W \| \| L \| A \| H \| R \| P \| W \| \| 98.3 \| 98.7 \| 100 \| 89.4 \| 100 \| 100 \| \| **989** \| **993** \| **1006** \| **899** \| **1006** \| **1006** \| |
| 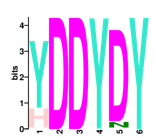 | \| 405 \| 406 \| 407 \| 408 \| 409 \| 410 \| \| --- \| --- \| --- \| --- \| --- \| --- \| \| Y \| D \| D \| Y \| D \| Y \| \| Y \| D \| D \| Y \| D \| Y \| \| 75.8 \| 100 \| 100 \| 99.7 \| 91.9 \| 100 \| \| **763** \| **1006** \| **1006** \| **1003** \| **925** \| **1006** \| |
| 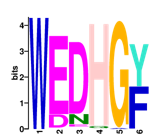 | \| 344 \| 345 \| 346 \| 347 \| 348 \| 349 \| \| --- \| --- \| --- \| --- \| --- \| --- \| \| W \| E \| D \| H \| G \| Y \| \| W \| E \| D \| H \| G \| F \| \| 99.8 \| 83.1 \| 81.4 \| 96.5 \| 97.6 \| 50 \| \| **1004** \| **836** \| **819** \| **971** \| **982** \| **503** \| |
| 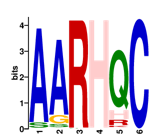 | \| 120 \| 121 \| 122 \| 123 \| 124 \| 125 \| \| --- \| --- \| --- \| --- \| --- \| --- \| \| A \| A \| R \| H \| Q \| C \| \| A \| A \| R \| H \| Q \| C \| \| 93.1 \| 84.1 \| 98.8 \| 98.9 \| 76 \| 99.5 \| \| **937** \| **846** \| **994** \| **995** \| **765** \| **1001** \| |
| 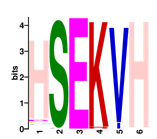 | \| 449 \| 450 \| 451 \| 452 \| 453 \| 454 \| \| --- \| --- \| --- \| --- \| --- \| --- \| \| H \| S \| E \| K \| V \| H \| \| H \| S \| E \| K \| V \| H \| \| 87.8 \| 98.6 \| 99.5 \| 97 \| 92.6 \| 98.1 \| \| **883** \| **992** \| **1001** \| **976** \| **932** \| **987** \| |
| 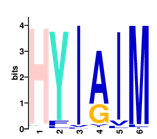 | \| 113 \| 114 \| 115 \| 116 \| 117 \| 118 \| \| --- \| --- \| --- \| --- \| --- \| --- \| \| H \| Y \| I \| A \| I \| M \| \| H \| Y \| I \| A \| I \| M \| \| 91.8 \| 90.4 \| 92.4 \| 67.8 \| 89.3 \| 92.2 \| \| **924** \| **909** \| **930** \| **682** \| **898** \| **928** \| |
| 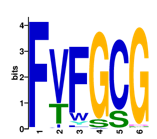 | \| 210 \| 211 \| 212 \| 213 \| 214 \| 215 \| \| --- \| --- \| --- \| --- \| --- \| --- \| \| F \| V \| F \| G \| C \| G \| \| F \| V \| F \| G \| C \| G \| \| 96.9 \| 66.8 \| 77.7 \| 87.5 \| 75.9 \| 93.5 \| \| **975** \| **672** \| **782** \| **880** \| **764** \| **941** \| |
| 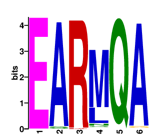 | \| 461 \| 462 \| 463 \| 464 \| 465 \| 466 \| \| --- \| --- \| --- \| --- \| --- \| --- \| \| E \| A \| R \| M \| Q \| A \| \| E \| A \| R \| M \| Q \| A \| \| 98.6 \| 96.1 \| 97.2 \| 70.8 \| 98.8 \| 95.6 \| \| **992** \| **967** \| **978** \| **712** \| **994** \| **962** \| |
| 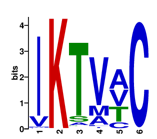 | \| 425 \| 426 \| 427 \| 428 \| 430 \| 430 \| \| --- \| --- \| --- \| --- \| --- \| --- \| \| I \| K \| T \| V \| A \| C \| \| I \| K \| T \| V \| A \| C \| \| 85.2 \| 99.1 \| 84.9 \| 67.2 \| 33.3 \| 98.3 \| \| **857** \| **997** \| **854** \| **676** \| **335** \| **989** \| |
| 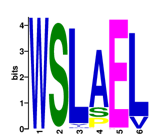 | \| 189 \| 190 \| 191 \| 192 \| 193 \| 194 \| \| --- \| --- \| --- \| --- \| --- \| --- \| \| W \| S \| L \| A \| E \| L \| \| W \| S \| L \| A \| E \| L \| \| 98.9 \| 96.9 \| 93 \| 71.2 \| 97.6 \| 86 \| \| **995** \| **975** \| **936** \| **716** \| **982** \| **865** \| |
| 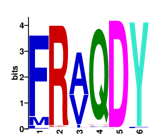 | \| 337 \| 338 \| 339 \| 340 \| 341 \| 342 \| \| --- \| --- \| --- \| --- \| --- \| --- \| \| F \| R \| A \| Q \| D \| Y \| \| F \| R \| A \| Q \| D \| Y \| \| 84.8 \| 92.3 \| 52.7 \| 92.6 \| 96 \| 94.1 \| \| **853** \| **929** \| **530** \| **932** \| **966** \| **947** \| |
| 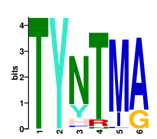 | \| 374 \| 375 \| 376 \| 377 \| 378 \| 379 \| \| --- \| --- \| --- \| --- \| --- \| --- \| \| T \| Y \| N \| T \| I \| A \| \| T \| Y \| N \| T \| M \| A \| \| 92.6 \| 92.1 \| 59.5 \| 82.4 \| 75.8 \| 74.3 \| \| **932** \| **927** \| **599** \| **829** \| **763** \| **747** \| |
| 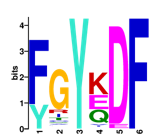 | \| 321 \| 322 \| 323 \| 324 \| 325 \| 326 \| \| --- \| --- \| --- \| --- \| --- \| --- \| \| F \| G \| Y \| E \| D \| F \| \| F \| G \| Y \| K \| D \| F \| \| 71.4 \| 65.6 \| 98.8 \| 38.5 \| 93.4 \| 98.4 \| \| **718** \| **660** \| **994** \| **387** \| **940** \| **990** \| |
| 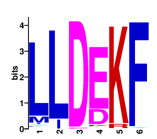 | \| 362 \| 363 \| 364 \| 365 \| 366 \| 367 \| \| --- \| --- \| --- \| --- \| --- \| --- \| \| L \| L \| D \| E \| K \| F \| \| L \| L \| D \| E \| K \| F \| \| 84.3 \| 89.8 \| 97.7 \| 74.8 \| 94 \| 97.3 \| \| **848** \| **903** \| **983** \| **752** \| **946** \| **979** \| |

| Non metazoan 6 residues wide |  |
| --- | --- |
| MEME discovered motif | Conservation (N=213) |
| 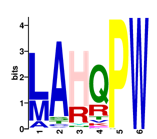 | \| 166 \| 167 \| 168 \| 169 \| 170 \| 171 \| \| --- \| --- \| --- \| --- \| --- \| --- \| \| L \| A \| H \| R \| P \| W \| \| L \| A \| H \| Q \| P \| W \| \| 61.5 \| 81.7 \| 68.1 \| 59.2 \| 97.7 \| 98.1 \| \| **131** \| **174** \| **145** \| **126** \| **208** \| **209** \| |
| 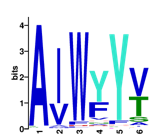 | \| 392 \| 393 \| 394 \| 395 \| 396 \| 397 \| \| --- \| --- \| --- \| --- \| --- \| --- \| \| A \| I \| W \| N \| Y \| I \| \| A \| I \| W \| Y \| Y \| V \| \| 84.5 \| 60.1 \| 77 \| 45.1 \| 89.7 \| 50.7 \| \| **180** \| **128** \| **164** \| **96** \| **191** \| **108** \| |
| 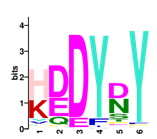 | \| 405 \| 406 \| 407 \| 408 \| 409 \| 410 \| \| --- \| --- \| --- \| --- \| --- \| --- \| \| Y \| D \| D \| Y \| D \| Y \| \| H \| D \| D \| Y \| D \| Y \| \| 41.3 \| 53.5 \| 80.3 \| 86.4 \| 31.5 \| 96.2 \| \| **88** \| **114** \| **171** \| **184** \| **67** \| **205** \| |
| 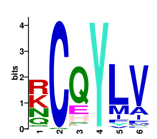 | \| 124 \| 125 \| 126 \| 127 \| 128 \| 129 \| \| --- \| --- \| --- \| --- \| --- \| --- \| \| Q \| C \| S \| Y \| L \| V \| \| R \| C \| Q \| Y \| L \| V \| \| 29.1 \| 90.1 \| 53.5 \| 91.5 \| 59.2 \| 59.6 \| \| **62** \| **192** \| **114** \| **195** \| **126** \| **127** \| |
| 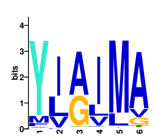 | \| 114 \| 115 \| 116 \| 117 \| 118 \| 119 \| \| --- \| --- \| --- \| --- \| --- \| --- \| \| Y \| I \| A \| I \| M \| A \| \| Y \| I \| A \| I \| M \| A \| \| 80.8 \| 48.4 \| 55.4 \| 58.7 \| 74.6 \| 70 \| \| **172** \| **103** \| **118** \| **125** \| **159** \| **149** \| |
| 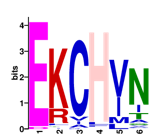 | \| 451 \| 452 \| 453 \| 454 \| 455 \| 456 \| \| --- \| --- \| --- \| --- \| --- \| --- \| \| E \| K \| V \| H \| V \| N \| \| E \| K \| C \| H \| V \| N \| \| 79.8 \| 59.6 \| 62.9 \| 71.8 \| 40.8 \| 44.1 \| \| **170** \| **127** \| **134** \| **153** \| **87** \| **94** \| |
| 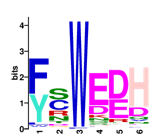 | \| 342 \| 343 \| 344 \| 345 \| 346 \| 347 \| \| --- \| --- \| --- \| --- \| --- \| --- \| \| Y \| T \| W \| E \| D \| H \| \| F \| S \| W \| E \| D \| H \| \| 52.6 \| 31.9 \| 92 \| 54.5 \| 51.2 \| 60.6 \| \| **112** \| **68** \| **196** \| **116** \| **109** \| **129** \| |
| 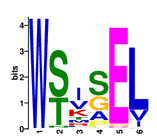 | \| 189 \| 190 \| 191 \| 192 \| 193 \| 194 \| \| --- \| --- \| --- \| --- \| --- \| --- \| \| W \| S \| L \| A \| E \| L \| \| W \| S \| I \| S \| E \| L \| \| 98.6 \| 55.9 \| 30 \| 41.8 \| 87.8 \| 72.8 \| \| **210** \| **119** \| **64** \| **89** \| **187** \| **155** \| |
| 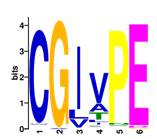 | \| 214 \| 215 \| 216 \| 217 \| 218 \| 219 \| \| --- \| --- \| --- \| --- \| --- \| --- \| \| C \| G \| I \| L \| P \| E \| \| C \| G \| I \| V \| P \| E \| \| 59.6 \| 88.3 \| 52.6 \| 36.2 \| 66.7 \| 90.1 \| \| **127** \| **188** \| **112** \| **77** \| **142** \| **192** \| |
| 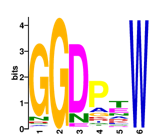 | \| 140 \| 141 \| 142 \| 143 \| 144 \| 145 \| \| --- \| --- \| --- \| --- \| --- \| --- \| \| G \| G \| D \| P \| E \| W \| \| G \| G \| D \| P \| T \| W \| \| 70.4 \| 87.8 \| 64.8 \| 44.6 \| 20.1 \| 97.7 \| \| **150** \| **187** \| **138** \| **95** \| **43** \| **208** \| |
| 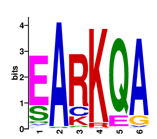 | \| 461 \| 462 \| 463 \| 464 \| 465 \| 466 \| \| --- \| --- \| --- \| --- \| --- \| --- \| \| E \| A \| R \| M \| Q \| A \| \| E \| A \| R \| K \| Q \| A \| \| 55.4 \| 89.2 \| 67.1 \| 76.5 \| 70.9 \| 70.9 \| \| **118** \| **190** \| **143** \| **163** \| **151** \| **151** \| |
| 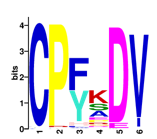 | \| 8 \| 9 \| 10 \|  \| 11 \| 12 \| \| --- \| --- \| --- \| --- \| --- \| --- \| \| C \| R \| A \| - \| E \| L \| \| C \| P \| F \| K \| D \| V \| \| 64.5 \| 87.4 \| 43 \| 22 \| 57.5 \| 53.3 \| \| **138** \| **187** \| **92** \| **47** \| **123** \| **114** \| |
| 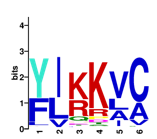 | \| 424 \| 425 \| 426 \| 427 \| 428 \| 429 \| \| --- \| --- \| --- \| --- \| --- \| --- \| \| Y \| I \| K \| T \| V \| A \| \| Y \| I \| K \| K \| V \| C \| \| 50.2 \| 51.6 \| 49.3 \| 63.8 \| 39.9 \| 47.4 \| \| **107** \| **110** \| **105** \| **136** \| **85** \| **101** \| |
| 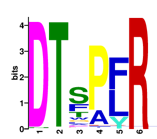 | \| 385 \| 386 \| 387 \| 388 \| 389 \| 390 \| \| --- \| --- \| --- \| --- \| --- \| --- \| \| D \| T \| S \| V \| L \| R \| \| D \| T \| S \| P \| F \| R \| \| 76.5 \| 75.6 \| 34.7 \| 62 \| 38 \| 80.8 \| \| **163** \| **161** \| **74** \| **132** \| **81** \| **172** \| |
| 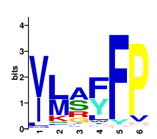 | \| 82 \| 83 \| 84 \| 85 \| 86 \| 87 \| \| --- \| --- \| --- \| --- \| --- \| --- \| \| V \| M \| G \| L \| H \| P \| \| I \| L \| A \| F \| F \| P \| \| 39 \| 70 \| 41.3 \| 39.4 \| 57.3 \| 82.6 \| \| **83** \| **149** \| **88** \| **84** \| **122** \| **176** \| |

| Metazoan 8 residues wide |  |
| --- | --- |
| MEME discovered motif | Conservation (N=1006) |
| 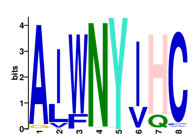 | \| 392 \| 393 \| 394 \| 395 \| 396 \| 397 \| 398 \| 399 \| \| --- \| --- \| --- \| --- \| --- \| --- \| --- \| --- \| \| A \| I \| W \| N \| Y \| I \| H \| C \| \| A \| I \| W \| N \| Y \| I \| H \| C \| \| 95.5 \| 68.3 \| 84 \| 97 \| 100 \| 74.5 \| 84.2 \| 92.7 \| \| **961** \| **687** \| **845** \| **976** \| **1006** \| **749** \| **847** \| **933** \| |
| 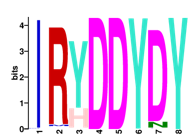 | \| 403 \| 404 \| 405 \| 406 \| 407 \| 408 \| 409 \| 410 \| \| --- \| --- \| --- \| --- \| --- \| --- \| --- \| --- \| \| I \| R \| Y \| D \| D \| Y \| D \| Y \| \| I \| R \| Y \| D \| D \| Y \| D \| Y \| \| 98.7 \| 95.1 \| 75.8 \| 100 \| 100 \| 99.7 \| 91.9 \| 100 \| \| **993** \| **957** \| **763** \| **1006** \| **1006** \| **1003** \| **925** \| **1006** \| |
| 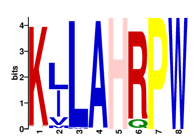 | \| 164 \| 165 \| 166 \| 167 \| 168 \| 169 \| 170 \| 171 \| \| --- \| --- \| --- \| --- \| --- \| --- \| --- \| --- \| \| K \| L \| L \| A \| H \| R \| P \| W \| \| K \| L \| L \| A \| H \| R \| P \| W \| \| 95.9 \| 40.7 \| 98.3 \| 98.7 \| 100 \| 89.4 \| 100 \| 100 \| \| **965** \| **409** \| **989** \| **993** \| **1006** \| **899** \| **1006** \| **1006** \| |
| 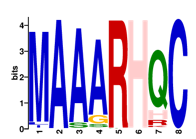 | \| 118 \| 119 \| 120 \| 121 \| 122 \| 123 \| 124 \| 125 \| \| --- \| --- \| --- \| --- \| --- \| --- \| --- \| --- \| \| M \| A \| A \| A \| R \| H \| Q \| C \| \| M \| A \| A \| A \| R \| H \| Q \| C \| \| 92.2 \| 99.1 \| 93.1 \| 84.1 \| 98.8 \| 98.9 \| 76 \| 99.5 \| \| **928** \| **997** \| **937** \| **846** \| **994** \| **995** \| **765** \| **1001** \| |
| 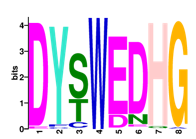 | \| 341 \| 342 \| 343 \| 344 \| 345 \| 346 \| 347 \| 348 \| \| --- \| --- \| --- \| --- \| --- \| --- \| --- \| --- \| \| D \| Y \| T \| W \| E \| D \| H \| G \| \| D \| Y \| S \| W \| E \| D \| H \| G \| \| 96 \| 94.1 \| 59.3 \| 99.8 \| 83.1 \| 81.4 \| 96.5 \| 97.6 \| \| **966** \| **947** \| **597** \| **1004** \| **836** \| **819** \| **971** \| **982** \| |
| 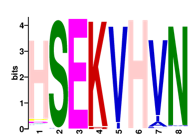 | \| 449 \| 450 \| 451 \| 452 \| 453 \| 454 \| 455 \| 456 \| \| --- \| --- \| --- \| --- \| --- \| --- \| --- \| --- \| \| H \| S \| E \| K \| V \| H \| V \| N \| \| H \| S \| E \| K \| V \| H \| V \| N \| \| 87.8 \| 98.6 \| 99.5 \| 97 \| 92.6 \| 98.1 \| 83.8 \| 94.6 \| \| **883** \| **992** \| **1001** \| **976** \| **932** \| **987** \| **843** \| **952** \| |
| 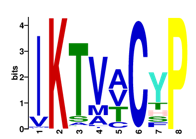 | \| 425 \| 426 \| 427 \| 428 \| 429 \| 430 \| 431 \| 432 \| \| --- \| --- \| --- \| --- \| --- \| --- \| --- \| --- \| \| I \| K \| T \| V \| A \| C \| Y \| P \| \| I \| K \| T \| V \| A \| C \| Y \| P \| \| 85.2 \| 99.1 \| 84.9 \| 67.2 \| 33.3 \| 98.3 \| 53.1 \| 99.9 \| \| **857** \| **997** \| **854** \| **676** \| **335** \| **989** \| **534** \| **1005** \| |
| 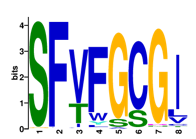 | \| 209 \| 210 \| 211 \| 212 \| 213 \| 214 \| 215 \| 216 \| \| --- \| --- \| --- \| --- \| --- \| --- \| --- \| --- \| \| S \| F \| V \| F \| G \| C \| G \| I \| \| S \| F \| V \| F \| G \| C \| G \| I \| \| 96.4 \| 96.9 \| 66.8 \| 77.7 \| 87.5 \| 75.9 \| 93.5 \| 71.8 \| \| **970** \| **975** \| **672** \| **782** \| **880** \| **764** \| **941** \| **722** \| |
| 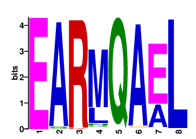 | \| 461 \| 462 \| 463 \| 464 \| 465 \| 466 \| 467 \| 468 \| \| --- \| --- \| --- \| --- \| --- \| --- \| --- \| --- \| \| E \| A \| R \| M \| Q \| A \| A \| L \| \| E \| A \| R \| M \| Q \| A \| E \| L \| \| 98.6 \| 96.1 \| 97.2 \| 70.8 \| 98.8 \| 95.6 \| 69.4 \| 98 \| \| **992** \| **967** \| **978** \| **712** \| **994** \| **962** \| **698** \| **986** \| |
| 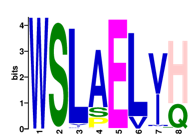 | \| 189 \| 190 \| 191 \| 192 \| 193 \| 194 \| 195 \| 196 \| \| --- \| --- \| --- \| --- \| --- \| --- \| --- \| --- \| \| W \| S \| L \| A \| E \| L \| I \| Q \| \| W \| S \| L \| A \| E \| L \| V \| H \| \| 98.9 \| 96.9 \| 93 \| 71.2 \| 97.6 \| 86 \| 52.9 \| 69.9 \| \| **995** \| **975** \| **936** \| **716** \| **982** \| **865** \| **532** \| **703** \| |
| 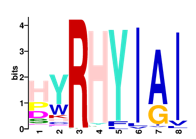 | \| 110 \| 111 \| 112 \| 113 \| 114 \| 115 \| 116 \| 117 \| \| --- \| --- \| --- \| --- \| --- \| --- \| --- \| --- \| \| S \| W \| R \| H \| Y \| I \| A \| I \| \| H \| Y \| R \| H \| Y \| I \| A \| I \| \| 43.2 \| 53.9 \| 97.5 \| 91.8 \| 90.4 \| 92.4 \| 67.8 \| 89.3 \| \| **435** \| **542** \| **981** \| **924** \| **909** \| **930** \| **682** \| **898** \| |
| 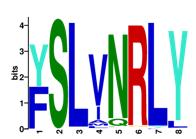 | \| 349 \| 350 \| 351 \| 352 \| 353 \| 354 \| 355 \| 356 \| \| --- \| --- \| --- \| --- \| --- \| --- \| --- \| --- \| \| Y \| S \| L \| I \| Q \| R \| L \| Y \| \| F \| S \| L \| V \| N \| R \| L \| Y \| \| 50 \| 98.5 \| 96.2 \| 68.6 \| 87.5 \| 94.1 \| 96.2 \| 89.9 \| \| **503** \| **991** \| **968** \| **690** \| **880** \| **947** \| **968** \| **904** \| |
| 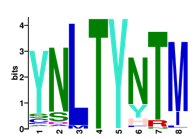 | \| 371 \| 372 \| 373 \| 374 \| 375 \| 376 \| 377 \| 378 \| \| --- \| --- \| --- \| --- \| --- \| --- \| --- \| --- \| \| Y \| S \| L \| T \| Y \| N \| T \| I \| \| Y \| N \| L \| T \| Y \| N \| T \| M \| \| 72.1 \| 71 \| 96 \| 92.6 \| 92.1 \| 59.5 \| 82.4 \| 75.8 \| \| **725** \| **714** \| **966** \| **932** \| **927** \| **599** \| **829** \| **763** \| |
| 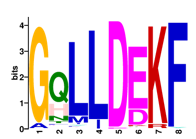 | \| 360 \| 361 \| 362 \| 363 \| 364 \| 365 \| 366 \| 367 \| \| --- \| --- \| --- \| --- \| --- \| --- \| --- \| --- \| \| G \| Q \| L \| L \| D \| E \| K \| F \| \| G \| Q \| L \| L \| D \| E \| K \| F \| \| 93.3 \| 54.9 \| 84.3 \| 89.8 \| 97.7 \| 74.8 \| 94 \| 97.3 \| \| **939** \| **552** \| **848** \| **903** \| **983** \| **752** \| **946** \| **979** \| |
| 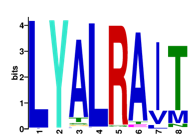 | \| 469 \| 470 \| 471 \| 472 \| 473 \| 474 \| 475 \| 476 \| \| --- \| --- \| --- \| --- \| --- \| --- \| --- \| --- \| \| L \| Y \| A \| L \| R \| A \| I \| T \| \| L \| Y \| A \| L \| R \| A \| I \| T \| \| 96.9 \| 97.6 \| 85.6 \| 95.7 \| 94.7 \| 90.4 \| 77 \| 75.7 \| \| **975** \| **982** \| **861** \| **963** \| **953** \| **909** \| **775** \| **762** \| |

| Non metazoan 8 residues wide |  |
| --- | --- |
| MEME discovered motif | Conservation (N=213) |
| 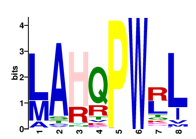 | \| 166 \| 167 \| 168 \| 169 \| 170 \| 171 \| 172 \| 173 \| \| --- \| --- \| --- \| --- \| --- \| --- \| --- \| --- \| \| L \| A \| H \| R \| P \| W \| L \| I \| \| L \| A \| H \| Q \| P \| W \| R \| L \| \| 61.5 \| 81.7 \| 68.1 \| 59.2 \| 97.7 \| 98.1 \| 31.9 \| 68.5 \| \| **131** \| **174** \| **145** \| **126** \| **208** \| **209** \| **68** \| **146** \| |
| 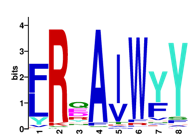 | \| 389 \| 390 \| 391 \| 392 \| 393 \| 394 \| 395 \| 396 \| \| --- \| --- \| --- \| --- \| --- \| --- \| --- \| --- \| \| L \| R \| R \| A \| I \| W \| N \| Y \| \| F \| R \| Q \| A \| I \| W \| Y \| Y \| \| 38 \| 80.8 \| 24.4 \| 84.5 \| 60.1 \| 77 \| 45.1 \| 89.7 \| \| **81** \| **172** \| **52** \| **180** \| **128** \| **164** \| **96** \| **191** \| |
| 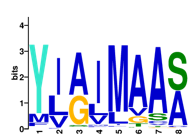 | \| 114 \| 115 \| 116 \| 117 \| 118 \| 119 \| 120 \| 121 \| \| --- \| --- \| --- \| --- \| --- \| --- \| --- \| --- \| \| Y \| I \| A \| I \| M \| A \| A \| A \| \| Y \| I \| A \| I \| M \| A \| A \| S \| \| 80.8 \| 48.4 \| 55.4 \| 58.7 \| 74.6 \| 70 \| 79.8 \| 50.2 \| \| **172** \| **103** \| **118** \| **125** \| **159** \| **149** \| **170** \| **107** \| |
| 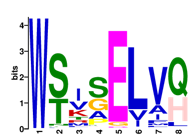 | \| 189 \| 190 \| 191 \| 192 \| 193 \| 194 \| 195 \| 196 \| \| --- \| --- \| --- \| --- \| --- \| --- \| --- \| --- \| \| W \| S \| L \| A \| E \| L \| I \| Q \| \| W \| S \| I \| S \| E \| L \| V \| Q \| \| 98.6 \| 55.9 \| 30 \| 41.8 \| 87.8 \| 72.8 \| 57.7 \| 54.5 \| \| **210** \| **119** \| **64** \| **89** \| **187** \| **155** \| **123** \| **116** \| |
| 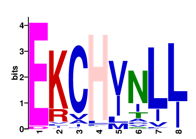 | \| 451 \| 452 \| 453 \| 454 \| 455 \| 456 \| 457 \| 458 \| \| --- \| --- \| --- \| --- \| --- \| --- \| --- \| --- \| \| E \| K \| V \| H \| V \| N \| L \| L \| \| E \| K \| C \| H \| V \| N \| L \| L \| \| 79.8 \| 59.6 \| 62.9 \| 71.8 \| 40.8 \| 44.1 \| 71.8 \| 59.2 \| \| **170** \| **127** \| **134** \| **153** \| **87** \| **94** \| **153** \| **126** \| |
| 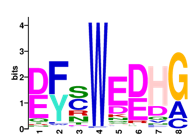 | \| 341 \| 342 \| 343 \| 344 \| 345 \| 346 \| 347 \| 348 \| \| --- \| --- \| --- \| --- \| --- \| --- \| --- \| --- \| \| D \| Y \| T \| W \| E \| D \| H \| G \| \| D \| F \| S \| W \| E \| D \| H \| G \| \| 45.1 \| 52.6 \| 31.9 \| 92 \| 54.5 \| 51.2 \| 60.6 \| 55.9 \| \| **96** \| **112** \| **68** \| **196** \| **116** \| **109** \| **129** \| **119** \| |
| 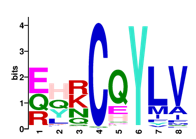 | \| 122 \| 123 \| 124 \| 125 \| 126 \| 127 \| 128 \| 129 \| \| --- \| --- \| --- \| --- \| --- \| --- \| --- \| --- \| \| R \| H \| Q \| C \| S \| Y \| L \| V \| \| E \| H \| R \| C \| Q \| Y \| L \| V \| \| 40.4 \| 32.4 \| 29.1 \| 90.1 \| 53.5 \| 91.5 \| 59.2 \| 59.6 \| \| **86** \| **69** \| **62** \| **192** \| **114** \| **195** \| **126** \| **127** \| |
| 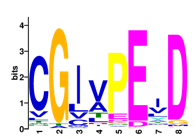 | \| 214 \| 215 \| 216 \| 217 \| 218 \| 219 \| 220 \| 221 \| \| --- \| --- \| --- \| --- \| --- \| --- \| --- \| --- \| \| C \| G \| I \| L \| P \| E \| G \| D \| \| C \| G \| I \| V \| P \| E \| I \| D \| \| 59.6 \| 88.3 \| 52.6 \| 36.2 \| 66.7 \| 90.1 \| 26.3 \| 82.2 \| \| **127** \| **188** \| **112** \| **77** \| **142** \| **192** \| **56** \| **175** \| |
| 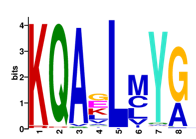 | \| 464 \| 465 \| 466 \| 468 \| 469 \| 470 \| 471 \| 472 \| \| --- \| --- \| --- \| --- \| --- \| --- \| --- \| --- \| \| M \| Q \| A \| L \| L \| Y \| A \| L \| \| K \| Q \| A \| L \| M \| Y \| G \| L \| \| 76.5 \| 70.9 \| 70.9 \| 89.2 \| 26.8 \| 74.6 \| 51.2 \| 83.6 \| \| **163** \| **151** \| **151** \| **190** \| **57** \| **159** \| **109** \| **178** \| |
| 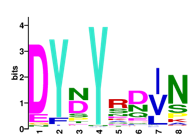 | \| 407 \| 408 \| 409 \| 410 \| 411 \| 412 \| 413 \| 414 \| \| --- \| --- \| --- \| --- \| --- \| --- \| --- \| --- \| \| D \| Y \| D \| Y \| G \| E \| V \| N \| \| D \| Y \| D \| Y \| R \| D \| I \| N \| \| 80.3 \| 86.4 \| 31.5 \| 96.2 \| 31.9 \| 36.6 \| 44.6 \| 49.3 \| \| **171** \| **184** \| **67** \| **205** \| **68** \| **78** \| **95** \| **105** \| |
| 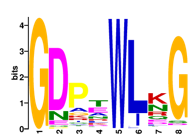 | \| 141 \| 142 \| 143 \| 143 \| 144 \| 146 \| 147 \| 148 \| \| --- \| --- \| --- \| --- \| --- \| --- \| --- \| --- \| \| G \| D \| P \| P \| E \| L \| L \| G \| \| G \| D \| P \| P \| T \| L \| K \| G \| \| 87.8 \| 64.8 \| 44.6 \| 44.6 \| 20.2 \| 76.5 \| 25.4 \| 75.6 \| \| **187** \| **138** \| **95** \| **95** \| **43** \| **163** \| **54** \| **161** \| |
| 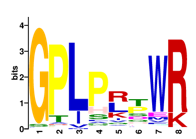 | \| 105 \| 106 \| 107 \| 108 \| 109 \| 111 \| 112 \| 114 \| \| --- \| --- \| --- \| --- \| --- \| --- \| --- \| --- \| \| G \| P \| L \| A \| S \| W \| R \| Y \| \| G \| P \| L \| P \| R \| W \| R \| Y \| \| 85.9 \| 75.1 \| 74.6 \| 54.5 \| 29.1 \| 70.4 \| 72.8 \| 80.8 \| \| **183** \| **160** \| **159** \| **116** \| **62** \| **150** \| **155** \| **172** \| |
| 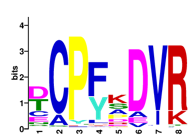 | \| 7 \| 8 \| 9 \| 10 \|  \| 11 \| 12 \| 13 \| \| --- \| --- \| --- \| --- \| --- \| --- \| --- \| --- \| \| E \| C \| R \| A \| - \| E \| L \| K \| \| D \| C \| P \| F \| K \| D \| V \| R \| \| 23.8 \| 64.5 \| 87.4 \| 43 \| 22 \| 57.5 \| 53.3 \| 58.4 \| \| **51** \| **138** \| **187** \| **92** \| **47** \| **123** \| **114** \| **125** \| |
| 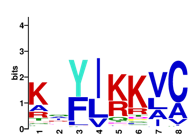 | \| 422 \| 424 \| 425 \| 426 \| 427 \| 428 \| 429 \| 430 \| \| --- \| --- \| --- \| --- \| --- \| --- \| --- \| --- \| \| K \| Y \| I \| K \| T \| V \| A \| C \| \| K \| Y \| I \| K \| K \| V \| C \| C \| \| 60.6 \| 50.2 \| 51.6 \| 49.3 \| 63.8 \| 39.9 \| 47.4 \| 38 \| \| **129** \| **107** \| **110** \| **105** \| **136** \| **85** \| **101** \| **81** \| |
| 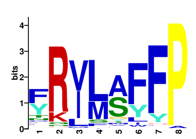 | \| 80 \| 81 \| 82 \| 83 \| 84 \| 85 \| 86 \| 87 \| \| --- \| --- \| --- \| --- \| --- \| --- \| --- \| --- \| \| A \| V \| V \| M \| G \| L \| H \| P \| \| F \| R \| I \| L \| A \| F \| F \| P \| \| 25.8 \| 60.1 \| 39 \| 70 \| 41.3 \| 39.4 \| 57.3 \| 82.6 \| \| **55** \| **128** \| **83** \| **149** \| **88** \| **84** \| **122** \| **176** \| |
